# SDC2 Stabilization by USP14 Promotes Gastric Cancer Progression through Co-option of PDK1

**DOI:** 10.7150/ijbs.84331

**Published:** 2023-07-09

**Authors:** Li You, Yi Dou, Yu Zhang, Hongwei Xiao, Hong Lv, Gong-Hong Wei, Dazhi Xu

**Affiliations:** 1 Department of Gastric Surgery, Fudan University Shanghai Cancer, Shanghai 200032, China.; 2 Department of Oncology, Shanghai Medical College, Fudan University, Shanghai 200032, China.; 3 Key Laboratory of Animal Embryo Engineering and Molecular Breeding of Hubei province, Institute of Animal Husbandry and Veterinary, Hubei Academy of Agricultural Science, Wuhan 430064, China.; 4 Department of Pathology, Fudan University Shanghai Cancer Center, Shanghai 200032, China.; 5 MOE Key Laboratory of Metabolism and Molecular Medicine and Department of Biochemistry and Molecular Biology of School of Basic Medical Sciences, and Fudan University Shanghai Cancer Center, Shanghai Medical College of Fudan University, Shanghai 200032, China.

**Keywords:** Gastric cancer, SDC2, PDK1, FGF2, USP14.

## Abstract

Gastric cancer (GC) is a common malignancy and remains the fourth-leading cause of cancer-related deaths worldwide. Oncogenic potential of SDC2 has been implicated in multiple types of cancers, yet its role and underlying molecular mechanisms in GC remain unknown. Here, we found that SDC2 was highly expressed in GC and its upregulation correlated with poor prognosis in GC patients. Depletion of SDC2 significantly suppressed the growth and invasive capability of GC cells, while overexpressing SDC2 exerts opposite effects. Combined bioinformatics and experimental analyses substantiated that overexpression of SDC2 activated the AKT signaling pathway in GC, mechanistically through the interaction between SDC2 and PDK1-PH domain, thereby facilitating PDK1 membrane translocation to promote AKT activation. Moreover, SDC2 could also function as a co-receptor for FGF2 and was profoundly involved in the FGF2-AKT signaling axis in GC. Lastly, we revealed a mechanism on the USP14-mediated stabilization of SDC2 that is likely to contribute to SDC2 upregulation in GC tissues. Furthermore, we showed that IU1, a potent USP14 inhibitor, decreased the abundance of SDC2 in GC cells. Our findings indicate that SDC2 functions as a novel GC oncogene and has potential utility as a diagnostic marker and therapeutic target for GC.

## Introduction

Gastric cancer (GC) is one of the most common malignancies and remains the fourth-leading cause of cancer-related deaths worldwide 1, 2. Each year, approximately one million patients are newly diagnosed with GC, and approximately seventy-eight thousand die from GC 1. Although significant progress has been made in the surgical and nonsurgical treatments for GC, the long-term prognosis of GC patients is still far from satisfactory 1, 3, 4. For many of these patients, the cancer is in advanced stages at their initial diagnosis, so there is a high rate of recurrence even after a radical treatment. Furthermore, due to the heterogeneity and complexity of GC, the efficacy of adjuvant chemo-radiotherapy and immunotherapy varies greatly among different GC patients, and development of resistance to these therapies inevitably culminates in death 5-7. Therefore, there is an urgent need to understand the pathogenesis and biological mechanisms underlying GC progression, in order to identify key oncogenes that can potentially be used as early diagnostic markers and therapeutic targets.

A diverse array of signaling pathways and molecular determinants contribute to the progression of GC. Among them, dysregulation of the PI3K-AKT pathway can empower tumor cell growth, invasion, and resistance to chemotherapy 3, 8, and thus plays important roles in GC progression. PDK1 is a crucial independent driver of the PI3K/AKT signaling pathway and functions as a pivotal kinase for AKT phosphorylation 9, 10. PDK1 contains a lipid-binding, C-terminus Pleckstrin Homology (PH) domain and a Kinase domain at its N-terminus 10. The phosphorylation of AKT by PDK1 occurs upon the membrane translocation of cytosolic PDK1 and AKT; in this process, PDK1 is bound to the cell membrane *via* its PH domain, and then its kinase domain phosphorylates AKT 9-11. Moreover, several growth factors, including insulin-like growth factor 1 (IGF-1), basic fibroblast growth factor 2 (FGF2), and epidermal growth factor (EGF), can greatly activate the PI3K-AKT pathway, thereby facilitating malignant behaviors of cancers 12-16.

Syndecan-2 (SDC2) is a single-pass type I transmembrane protein that belongs to the four-member proteoglycan-syndecan family 17, 18. Gastrointestinal cancer cells specifically present methylated SDC2 regardless of the clinical stage, and assessment of DNA-based-SDC2 methylation in stool samples is a useful non-invasive method for early detection of colorectal cancer (CRC) 19, and more recently for GC 20. On the other hand, the biological role of SDC2 in human diseases was elusive until 2001, when a study reported that SDC2 increased the migratory behavior of CRC cells 21. Since then, several studies have characterized the biological functions of SDC2 in other tumors 22-27. For example, studies of lung adenocarcinoma, lung fibrosarcoma, as well as cancers of pancreas and breast found that SDC2 promoted cell invasion, tumor growth, and/or immune evasion 22, 24-27. Another study on osteosarcoma found that SDC2 increased chemotherapy-induced apoptosis 23. SDC2 also functions in the pathogenesis of non-cancer diseases, in that it regulates angiogenesis in rheumatoid arthritis and modulates chondrocyte differentiation in osteoarthritis 28, 29. Moreover, SDC2 is a membrane protein that acts as a co-receptor for specific heparin-binding growth factors, including vascular endothelial growth factor-A (VEGFA), FGF2, and hepatocyte growth factor (HGF) 30-32. SDC2 therefore mediates the downstream intracellular signaling pathways that are triggered by these growth factors. However, we know very little about the biological role and underlying molecular mechanisms of SDC2 in GC.

In this study, we examined the oncogenic role of SDC2 in the progression of GC and the relationship of its expression with the long-term prognosis of GC patients. We also assessed the biological effects of SDC2 overexpression and depletion, its physical interactions with PDK1 and its involvement in FGF2/PI3K/AKT signaling in GC cells. Inspired by recent studies that reported ubiquitin-mediated proteolysis of key signaling proteins 33-36 and our finding of physical interaction between SDC2 and ubiquitin-specific protease 14 (USP14), we evaluated the effect of USP14 in regulating the abundance of SDC2 in GC, and assessed the possible therapeutic utility of IU1, a small-molecule inhibitor that increases the ubiquitination-mediated degradation of SDC2 by targeting USP14.

## Materials and Methods

### Cell lines and cell culture

Two lines of human gastric cancer cells (AGS and HGC-27) were purchased from Procell Life Science & Technology Co., Ltd (Wuhan, China), and the other GC cell lines (GT38 and SNU-719) were purchased from Honsun Biological Technology Co., Ltd (Shanghai, China). AGS cells were cultured in Hams' F12 medium (Shanghai BasalMedia Technologies Co., Ltd.) that was supplemented with 10% fetal bovine serum (FBS; ExCell Bio. Jiangsu, China) and 1% penicillin-streptomycin (Shanghai BasalMedia Technologies Co., Ltd.). HGC-27, SNU719, and GT38 cells were cultured in RPMI-1640 medium (Shanghai BasalMedia Technologies Co., Ltd.) that was supplemented with 10% FBS and 1% penicillin-streptomycin. All cells were cultured in a humidified incubator with 5% carbon dioxide (Thermo Fisher Scientific, USA).

### Cell transfection

For transient transfection experiments, FLAG-tagged-*SDC2,* HA-tagged-*PDK1* recombinant constructs, HA-tagged-*Ub, or USP14* was transfected into HEK-293T cells for 48 h. For stable transfection experiments, virus packaging and infection were performed as previously described 37. Information for plasmids used in this study was present in **Table S1**.

### Migration and invasion assay

Migration and invasion assays were performed using transwell chambers (Corning Inc. USA). GC cells were plated in the upper chamber with 200 µL of low serum medium (2% FBS) F12 or 1640 medium at a density of 30,000 to 90,000 cells per well. Then, a medium containing 13% FBS was added to the lower chamber to induce chemo-attraction. After culturing for 18 h, cells in the lower chamber were fixed with 4% paraformaldehyde and stained with crystal violet. Five randomly-selected fields were photographed and counted for each treatment.

### Viability and proliferation assays

The Cell Counting Kit 8 (CCK8; Dojingdo, Japan) was used to measure cell viability and proliferation. Briefly, GC cells were seeded at a density of 1200 to 3000 cells per well in complete medium. A combination of 10 μL of CCK8 solution and 80 μL of serum-free medium was added into each well at 24 h, 48 h, 72 h, or 96 h after seeding. Culturing was then performed for 1.5 h, followed by measurement of absorbance at 450 nm.

### Western blotting and co-immunoprecipitation

Cells were lysed in RIPA lysis buffer (Shanghai Epizyme Biomedical Technology Co., Ltd, China) that was supplemented with a protease inhibitor cocktail and a phosphatase inhibitor cocktail (Bimake, USA). The protein concentration in the cell lysates was measured using the BCA protein assay kit (Solabrio Life Science, China). After separation and transfer of proteins to PVDF membranes, the membranes were blocked in 5% Tris-buffered bovine serum albumin with Tween 20 (TBST) for 1.5 h at room temperature, and the primary antibodies were then added (**Table S2**).

For Co-IP experiments, cells were lysed in a RIPA-weak lysis buffer (1% NP-40 and 0.25% sodium deoxycholate) and processed with a protease and phosphatase inhibitor cocktail. Then, cell lysates were immunoprecipitated with Anti-FLAG Magnetic Beads (Beyotime Biotechnology, China) at 4 °C overnight. The precipitates were washed with lysis buffer three times, and the attached proteins were eluted after boiling in an SDS-PAGE loading buffer.

### LC-MS analysis

HEK293T cells were transfected with a control plasmid or a FLAG-tagged-*SDC2* plasmid. Protein extracts were then incubated with 80 μL of anti-FLAG magnetic beads (Beyotime Biotechnology, China) for 12 h. The boiled immune-precipitated proteins were separated using SDS-PAGE, and different proteins were obtained by cutting the designated bands from the gel, which were then analyzed using a LC-MS system (Pujing Technology Co., LTD, Shanghai). Peptides and proteins were identified by using FragPipe version 1.8.

### Isolation of membrane proteins

Plasma membrane proteins were isolated using a membrane and cytoplasmic protein extraction kit (Keygen Biotech Corp., Ltd, China). First, cells were suspended in 1 mL of lysis buffer that was supplemented with a protease and phosphatase inhibitor cocktail, and cells were then incubated on ice for 40 min, with brief vortexing every 8 min followed by rapid return to ice. After separation by centrifugation (12,000 rpm for 10 min at 4 °C), the supernatant was removed, and the pellet was resuspended in 300 μL of pre-chilled extraction buffer. The pellet lysate was then incubated on ice for 30 min, with vortexing every 6 min followed by rapid return to ice. After centrifugation (12,000 rpm for 10 min at 4 °C), the supernatant (which contained plasma membrane proteins) was used for analysis.

### Extraction of lipid rafts

Lipid rafts were extracted using the Minute^TM^ Plasma Membrane-Derived Lipid Raft Isolation Kit (Invent, USA). Cells were suspended in 500 μL of Buffer A that was supplemented with a protease and phosphatase inhibitor cocktail for 35 min, with vigorous vortexing every 7 min and rapid return to ice, according to manufacturer's instructions. The cell suspension was then transferred into filter cartridges, which were centrifuged at 16,000 *g* for 30 s to disrupt the cell membranes. The suspension was then transferred into microfuge tubes and centrifuged at 1900 *g* for 5 min, and the supernatants were transferred into fresh microfuge tubes for second round of centrifugation at 3000 *g* for 15 min. The pellets were then were re-suspended in 400 μL of pre-chilled Buffer B and incubated on ice for 40 min, with brief vortexing every 10 min. Then, 400 μL of Buffer C was added at room-temperature. The sample was then centrifuged at 10,000 *g* for 2 min at 4 °C, and the lipid rafts that were floating on top were used for experiments.

### Immunofluorescence staining

A total of 40,000 GC cells were plated on a confocal dish (NEST Biotechnology Co.LTD, China) and cultured in a carbon dioxide incubator for 48 h. Then, cells were fixed in 4% paraformaldehyde, permeabilized in 0.5% Triton X-100, and blocked in a BSA buffer with 3% PBS for 1 h. The fixed cells were incubated with an SDC2 antibody (1:100, Santa Cruz, sc-365624) and a PDK1 antibody (1:200, Abcam, ab186870) at 4 °C overnight, and then stained with a secondary antibody (Jackson ImmunoResearch, USA) for 1 h at room temperature. The antibody-stained cells were examined using a fluorescence microscope (Leica Camera, Wetzlar, Germany).

### Enzyme-linked immunosorbent assay

The plasma membrane concentration of PIP3 and the concentration of FGF2 in the cell culture medium were measured using an enzyme-linked immunosorbent assay (ELISA) according to the manufacturer's protocol (Cloud-Clone, China). First, 50 µL of a standard or sample was added to each well of a 96-well plate, and 50 µL of Detection Reagent A was then added immediately. After incubation for 1 h at 37 °C, each well was washed 3 times, and then 100 µL of Detection Reagent B was added. After an additional 30 min of incubation, the solution in each well was discarded. Then, each well was washed 5 times and cultured with the Substrate Solution for 20 min, followed by measurement of absorbance at 450 nm.

### Flow cytometry

The protein level of SDC2 in the plasma membrane was also measured using flow cytometry with an SDC2 APC-conjugated antibody (10 µL/10^6^ cells, R&D systems, FAB2965A).

### GC xenografts in nude mice

All animal experiment were approved by the Ethics Committee of Fudan University Shanghai Cancer Center. First, 3 × 10^6^ HGC-27 cells or 2.5 × 10^6^ SNU-719 cells were transfected with a MISSION^®^ shRNA Plasmid DNA Control Vector (Sh-Control) or Sh-*SDC2*. The cells were suspended in a combination of 50 μL prechilled PBS and 50 μL Matrigel (Corning, USA), and then administered by subcutaneous injection into the right dorsal flank of BALB/c nude male mice that were 5 to 6 weeks old. Tumors were measured using a vernier caliper, and volume (mm^3^) was estimated as (length × width^2^)/2. All mice were sacrificed on day-27 or when the tumor volume reached 2000 mm^3^.

### Patient cohorts and clinical samples

Clinical data were used from two patient cohorts. For the first cohort, data were from our previous study that performed whole-genome sequencing and transcriptome sequencing for 50 GC specimens obtained from patients who received radical gastrectomy at Sun Yat-sen University Cancer Center (***SYSUCC Cohort***) from September 2014 to April 2018 38 (**Table S3 and S4**). For the second cohort, 108 clinical specimens (with paired cancerous and adjacent normal tissues) were obtained from GC patients who received gastrectomy at the Gastric Surgery Department, Fudan University Shanghai Cancer Center from March 2017 to November 2017 (***FUSCC cohort***, **Table S5**).

None of the patients in either cohort received neoadjuvant chemo-radiotherapy or immunotherapy, and all patients received follow-up for at least 5 years. These experiments were approved by the Ethics Committees of our institution, and written informed consent was provided by all patients.

### Immunohistochemistry

Paraffin-embedded tissue samples were obtained from the Department of Pathology, Fudan University Shanghai Cancer Center. IHC analyses were performed for SDC2 (1:50, R&D, MAB29651), PDK1 (1:100, Santa Cruz, sc-17765), p-AKT (1:40, CST, Ser473), Ki-67(1:400, CST, 8D5), and USP14 (1:100, Santa Cruz, sc-398009). IHC staining scores were calculated as previously described 37.

### Statistical analysis

The results from different groups were compared using Student's *t*-test (two groups) or a one-way analysis of variance (three or more groups). Factors influencing long-term survival were determined by univariate and multivariate Cox proportional hazards regression analysis. SPSS version 22.0 was used for statistical analyses. Data were expressed as means ± SDs, and a *P* value less than 0.05 was considered significant (*P < 0.05 and **P < 0.01).

## Results

### SDC2 is highly expressed in GC and its upregulation correlates with poor prognosis of GC patients

**Figure 1:**
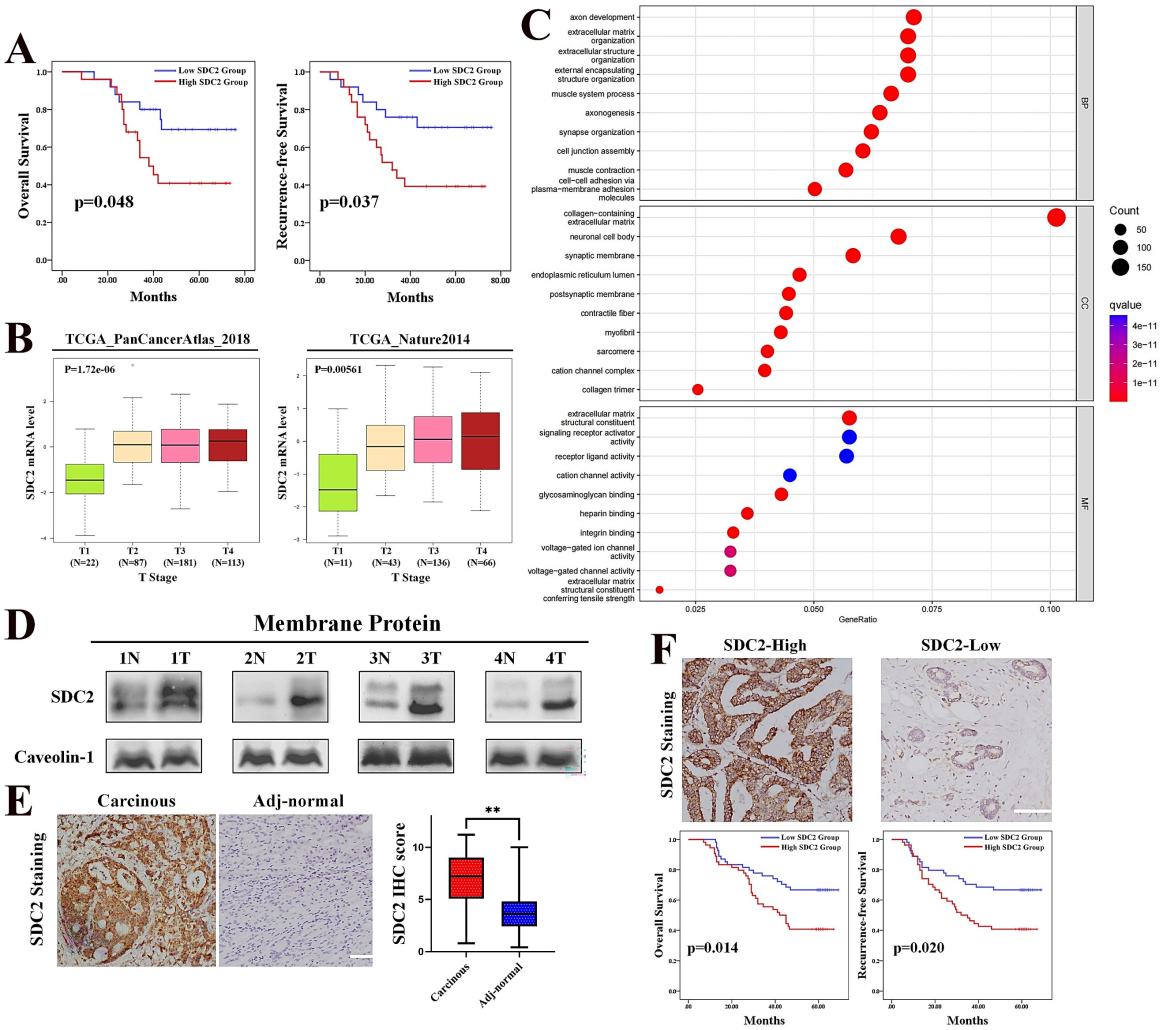
**Upregulation of SDC2 correlates with poor prognosis in patients with GC. A.** Overall survival and recurrence free survival in patients with low levels (N = 25) and high levels (N = 25) of SDC2 mRNA in GC tissues (SYSUCC cohort). **B.** SDC2 mRNA levels in specimens from two populations of patients with different T stages of GC (TCGA). **C.** Biological process (top), cellular components (middle), and molecular functions (bottom) related to SDC2 in GC (GO analysis). **D.** Expression of SDC2 in tumor tissues (T) and matched adjacent normal tissues (N) of four patients with GC (Western blotting). **E.** Representative images of SDC2 staining in GC tissue and adjacent normal tissue (Immunohistochemistry, scale bar: =100 µm). **F.** SDC2 expression, overall survival, and recurrence-free survival in GC patients with high expression (N = 54) and low expression (N = 54) of SDC2 in tumor tissues (Immunohistochemistry, FUSCC cohort).

Whole genome sequencing analysis of the SYSUCC cohort of GC patients (N = 50) demonstrated that SDC2 gene had a high frequency of mutation (16%). However, no significant differences have been observed in SDC2 expression level, long-term prognosis, or major clinicopathological parameters compared GC patients with wild type (SDC2-WT) to those with SDC2 mutation (SDC2-MT) (**Fig. S1A and S1B; Table S3**), suggesting that SDC2 mutation was likely to be a passenger event 39. In contrast, we observed that the GC patients whose tumors expressed higher levels of SDC2 (mRNA level > 8.12) had significantly shorter overall survival and recurrence-free survival compared to GC patients whose tumors had lower SDC2 expression (**Fig. 1A**). Consistently, the GEPIA2 cohort of GC patients with tumors that had high SDC2 expression indicated a significantly shorter OS time than those with low SDC2 expression (**Fig. S1C**) 40. These results suggested that upregulation of SDC2 might contribute to the progression of GC.

We next evaluated the correlation of SDC2 mRNA level with the clinical stages in GC patients by analyzing two independent TCGA GC cohorts. The results indicated that GC patients with higher T stages had significantly higher expression of SDC2 compared to those with lower T stages (**Fig. 1B**). Moreover, the GC patients with higher N stages also had a higher expression of SDC2 compared to those with low N stages and primary GC tumors without lymph node metastasis (**Fig. S1D**). These results led to the hypothesis that SDC2 may have an oncogenic and clinically relevant role in GC.

SDC2 is a transmembrane protein that usually functioned in a form of dimer or oligomer 24, and has a signaling peptide at the region of 1~15aa. In line with this, gene ontology (GO) enrichment analysis showed that the molecular functions of SDC2 in GC strongly correlated with “signaling receptors activator activity” and “receptor ligand activity” (**Fig. 1C**), suggesting that SDC2 might modulate cytokine-induced intracellular signaling pathways in GC. Western blotting showed that the protein levels of SDC2 were higher in tumor specimens than in matched adjacent normal tissues (**Fig. 1D and 1E**). Moreover, GC patients in the FUSCC cohort whose tumors had increased SDC2 IHC staining (IHC score ≥ 7.2) had a significantly shorter postoperative survival time (**Fig. 1F**). These findings demonstrated significant correlations of SDC2 expression with the aggressiveness of GC tumors and poor clinical outcomes in GC patients.

### SDC2 promotes proliferation and invasive capability of GC cells and its upregulation correlates with activation of the PI3K/AKT pathway

**Figure 2:**
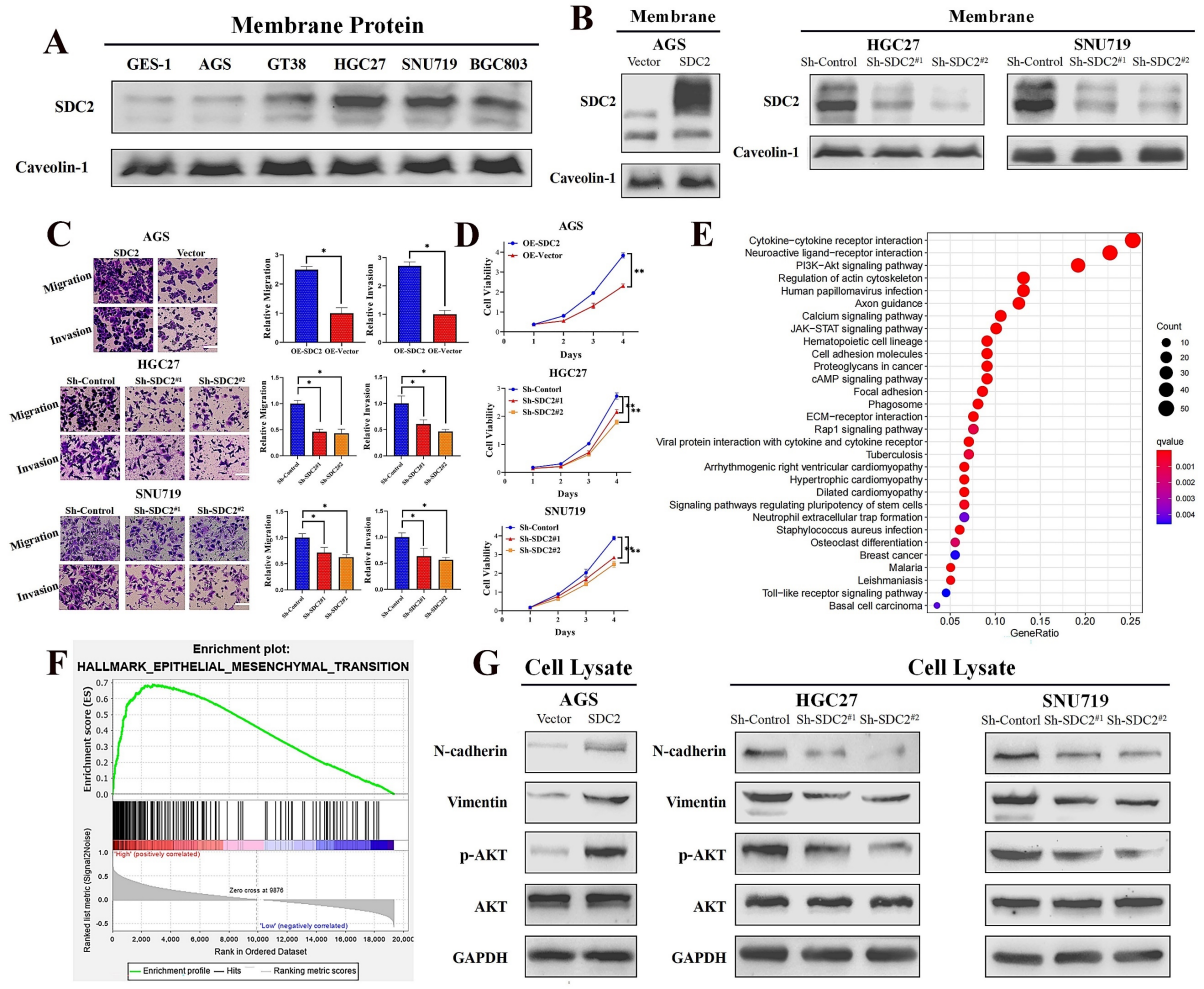
**SDC2 promotes proliferation and invasiveness of GC cells and its upregulation correlates with the activation of AKT pathway. A.** Protein levels of SDC2 in five lines of GC cells and one line of normal gastric epithelial cells (GES-1) (Western blotting). **B.** Protein levels of SDC2 in three lines of GC cells after stable transfection with plasmids that promoted overexpression (OE-SDC2) or knockdown (shRNA) of SDC2 (Western blotting). **C & D.** Migration, invasion, and proliferation of three lines of GC cells that were transfected with different plasmids (Transwell assay, CCK8 assay. Scale bar: =100 µm). **E & F.** SDC2-associated signaling pathways in GC based on pathways and gene sets (KEGG Enrichment and Hallmark Analysis). **G.** Protein level of mesenchymal markers (N-cadherin, Vimentin) and p-AKT, in three lines of GC cells that were transfected with different SDC2 plasmids (Western blotting).

Next, we investigated the functional significance of SDC2 in GC by examining its effect on cell growth and invasive ability. We first applied western blotting to examine the protein levels of SDC2 in five GC cell lines (AGS, GT38, HGC27, SNU719, and BGC803) and one normal gastric epithelial cell line (GES-1). Compared to the normal (GES-1) cells, there was markedly higher protein expression of SDC2 in HGC-27, SNU-719, and BGC-803 cells (**Fig. 2A**). We then established an AGS cell model that had overexpression of SDC2, and SNU-719 and HGC-27 cell models with knockdown of SDC2 (**Fig. 2B; Fig. S2A**). SDC2 knockdown significantly inhibited cell invasive ability (based on the transwell assay) and attenuated cell proliferation (based on the CCK8 assay); while SDC2 over-expression led to increased cell invasiveness and cell proliferation (**Fig. 2C and 2D; Fig. S2B**).

To explore the possible molecular mechanisms of SDC2 and its global correlation with potential signaling pathways, we applied Kyoto Encyclopedia of Genes and Genomes (KEGG) enrichment analysis 41 and a gene set enrichment analysis (GSEA) of 50 hallmark pathways 42. These analyses used our GC tumor transcriptomics data from the SYSUCC cohort, with stratification into groups that had high or low expression of SDC2. The results showed that SDC2 upregulation correlates with activation of the PI3K/AKT signaling pathway (**Fig. 2E**) and upregulation of the EMT-related gene signature (**Fig. 2F**). Consistently, KEGG enrichment analysis of TCGA GC gene profiling data also indicated that high expression of SDC2 was apparently associated with the PI3K/AKT signaling pathway (**Fig. S2C**). In line with the pathway analysis, western blotting results showed that SDC2 knockdown attenuated the phosphorylation of AKT and the expression of mesenchymal markers (vimentin and N-cadherin), whereas SDC2 overexpression increased AKT phosphorylation and the expression of these mesenchymal markers (**Fig. 2G**). These results demonstrated that SDC2 overexpression greatly promoted the proliferation and invasiveness of GC cells, induced the PI3K/AKT signaling pathway, and increased the expression of genes that function in the EMT. All of these effects could contribute to the progression of GC.

### SDC2 promotes GC tumor growth in vivo

**Figure 3:**
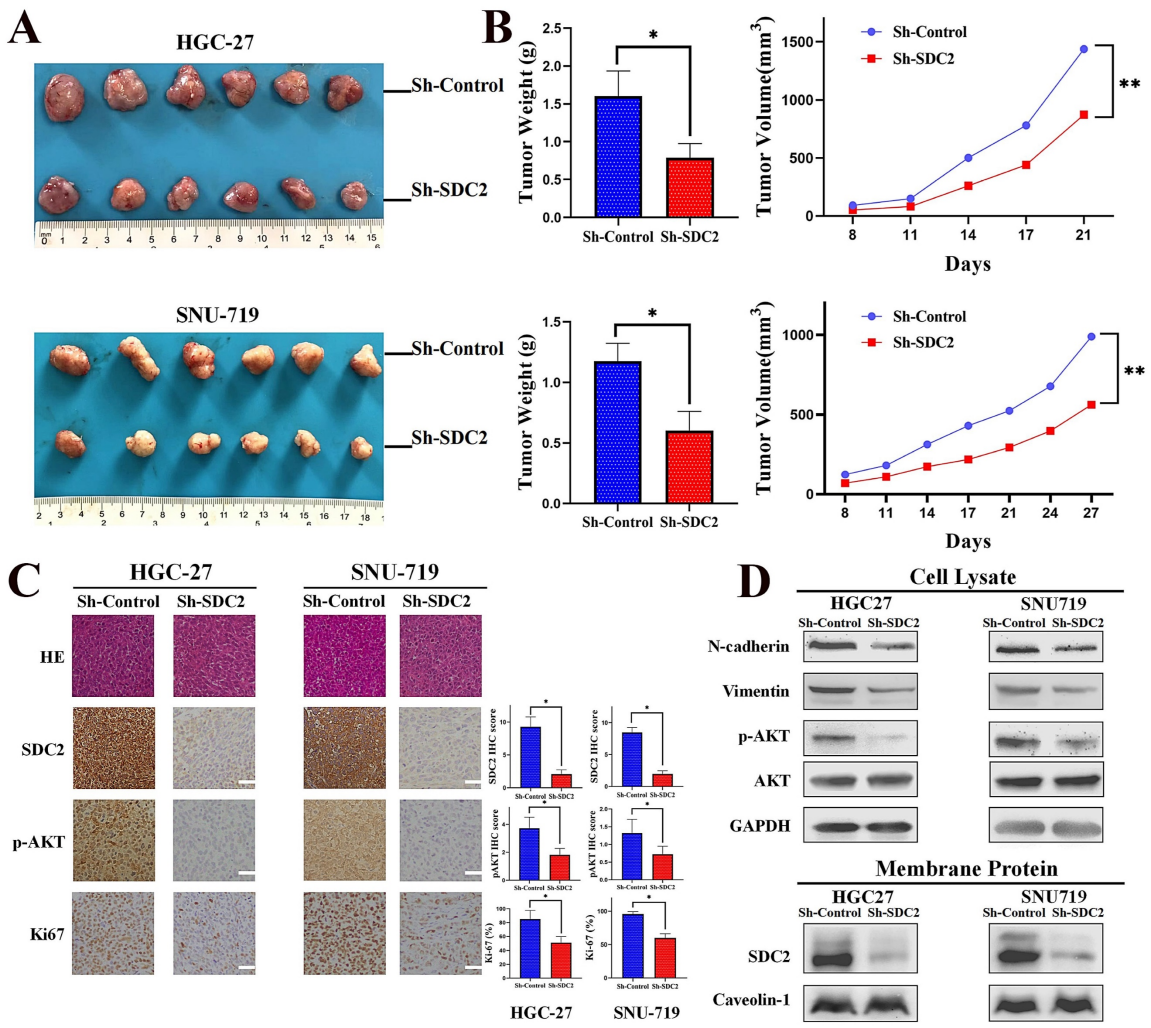
** SDC2 promotes GC tumor growth in vivo. A & B.** Morphology, weight, and average volume of subcutaneous tumors formed by two lines of GC cells that were transfected with different plasmids (Xenograft transplantation, N=6).** C.** Staining for SDC2, pAKT, and Ki67 in tumors from the two xenograft models that were transfected with different plasmids (Immunohistochemistry, scale bar: 50 µm). **D.** Protein levels of N-cadherin, vimentin, pAKT, AKT, and SDC2 in tumors from the two xenograft models that were transfected with different plasmids (Western blotting).

We then examined the effects of SDC2 on GC tumor progression *in vivo* by establishment of tumor xenograft mouse models using SNU-719 and HGC-27 cells with or without SDC2 knockdown. Measurements of the temporal changes of tumors showed that SDC2 knockdown reduced the growth, weight, and volume of tumors in both xenograft mouse models (**Fig. 3A and 3B**). We then performed IHC staining to measure the expression of SDC2, p-AKT, and Ki67 in tumor samples from the xenograft models. The results demonstrated that SDC2 knockdown led to significantly lower IHC scores for p-AKT and Ki67 in both models (**Fig. 3C**)*.* The western blotting results confirmed that SDC2 knockdown decreased the levels of p-AKT, vimentin, and N-cadherin in tumors (**Fig. 3D**). Altogether, these data demonstrated that depletion of SDC2 suppressed the growth of GC tumor xenografts *in vivo*.

### SDC2 promotes membrane translocation of PDK1 by interacting with its PH domain

**Figure 4:**
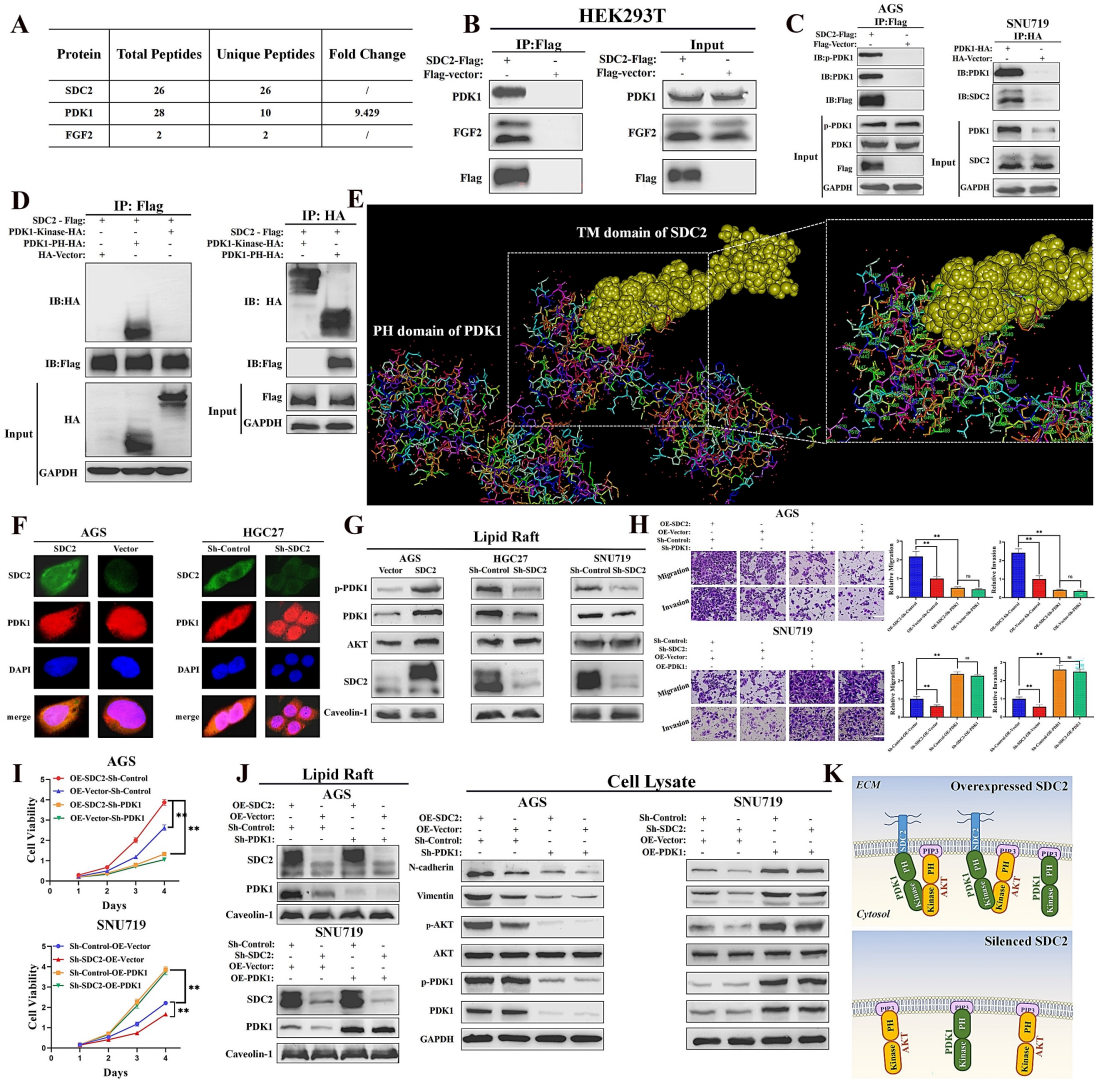
** SDC2 facilitates the membrane translocation of PDK1 by interacting with its PH domain in GC cells. A & B.** Association of PDK1 and FGF2 with SDC2 (LC/MS analysis, Co-IP assay).** C.** Interaction of SDC2 and PDK1 in GC cells (Co-IP assays).** D & E.** Interaction of SDC2 and the PH domain of PDK1 (Co-IP assays, using HEK293T cells; molecular docking performed by Discovery Studio^TM^). **F.** Colocalization of SDC2 and PDK1 in AGS and HGC-27 cells (Double immunofluorescent staining).** G.** Effect of SDC2 on membrane translocation of PDK1 (Western blotting of lipid raft).** H, I, & J.** Dependence of the oncogenic function of SDC2 on the PDK1-AKT signaling pathway in GC cells (CCK8 assay, Transwell assay, and Western blotting. Scale bar: =100 µm). **K.** A schematic diagram showing the proposed mechanism.

We next explored the possible mechanisms underlying the functional roles of SDC2 in GC severity and tumor progression. Thus, we identified SDC2-associated proteins via a combined co-immunoprecipitation (Co-IP) and LC-MS analysis. The results showed that PDK1 and FGF2 were physically associated with SDC2 (**Fig. 4A and 4B; Fig. S3A**). We also examined the concentration of FGF2 in the culture medium of HEK293T cells that were transfected with SDC2-Flag or Flag-vector plasmids (**Fig. S3B**). The Co-IP and western blotting results confirmed an interaction between SDC2 and PDK1 in GC cells (**Fig. 4C**). We then examined the domain(s) of PDK1 that potentially bind to SDC2 by analyzing the interactions between SDC2 and fragmented PDK1 recombinant proteins using Co-IP assays. The results showed that the PH domain was required for SDC2 binding (**Fig. 4D**). Furthermore, the molecular docking results from Discovery Studio™ supported the presence of an interaction between the PH domain of PDK1 and SDC2. Analysis using available structures in the Protein Data Bank (PDB) also demonstrated the protein-protein docking between the PH domain of PDK1 (1W1H) and the transmembrane domain of SDC2 (6ITH) (**Fig. 4E**). The cytoplasmic and extracellular domains of SDC2 have not yet been resolved based on the PDB query. Thus, further research is needed to analyze the tertiary structure of the entire SDC2 protein and the nature of its interaction with PDK1.

Because SDC2 is a membrane protein, we next investigated whether the interaction between PDK1 and SDC2 could affect the membrane location of PDK1. Immunofluorescence assays confirmed a co-localization of PDK1 and SDC2 in the plasma membrane and cytosol of AGS and HGC-27 cells (**Fig. 4F**). In addition, western blotting analysis of lipid rafts (**Fig. 4G**) and membrane proteins (**Fig. S3C**) demonstrated that overexpression of SDC2 promoted the membrane translocation of PDK1, while SDC2 knockdown attenuated this effect.

Previous studies established that PDK1 plays a vital role in activation of the AKT pathway and the phosphorylation of AKT, and that PDK1 knockdown inhibited the PDK1-AKT signaling pathway, whereas PDK1 upregulation significantly promoted this pathway 9, 10. We thus evaluated the effect of the AKT pathway in modulating the oncogenic functions of SDC2 in GC cells. The results of the transwell and CCK8 assays demonstrated that suppressing the PDK1-AKT pathway significantly reversed the oncogenic effects of SDC2-overexpression in AGS cells. SDC2 knockdown in SNU-719 cells suppressed cell growth and migration, but upregulation of the PDK1-AKT pathway reversed this effect (**Fig. 4H and 4I**). These results were consistent with the western blotting results (**Fig. 4J**) and with our interpretation that the oncogenic effect of SDC2 depends on the PDK1-AKT pathway. Taken together, these results suggested that SDC2 activates the AKT pathway by facilitating the membrane translocation of PDK1, whose PH domain interacts with SDC2 (**Fig. 4K**).

### High expression of SDC2 and PDK1 is associated with shorter postoperative survival of GC patients

**Figure 5:**
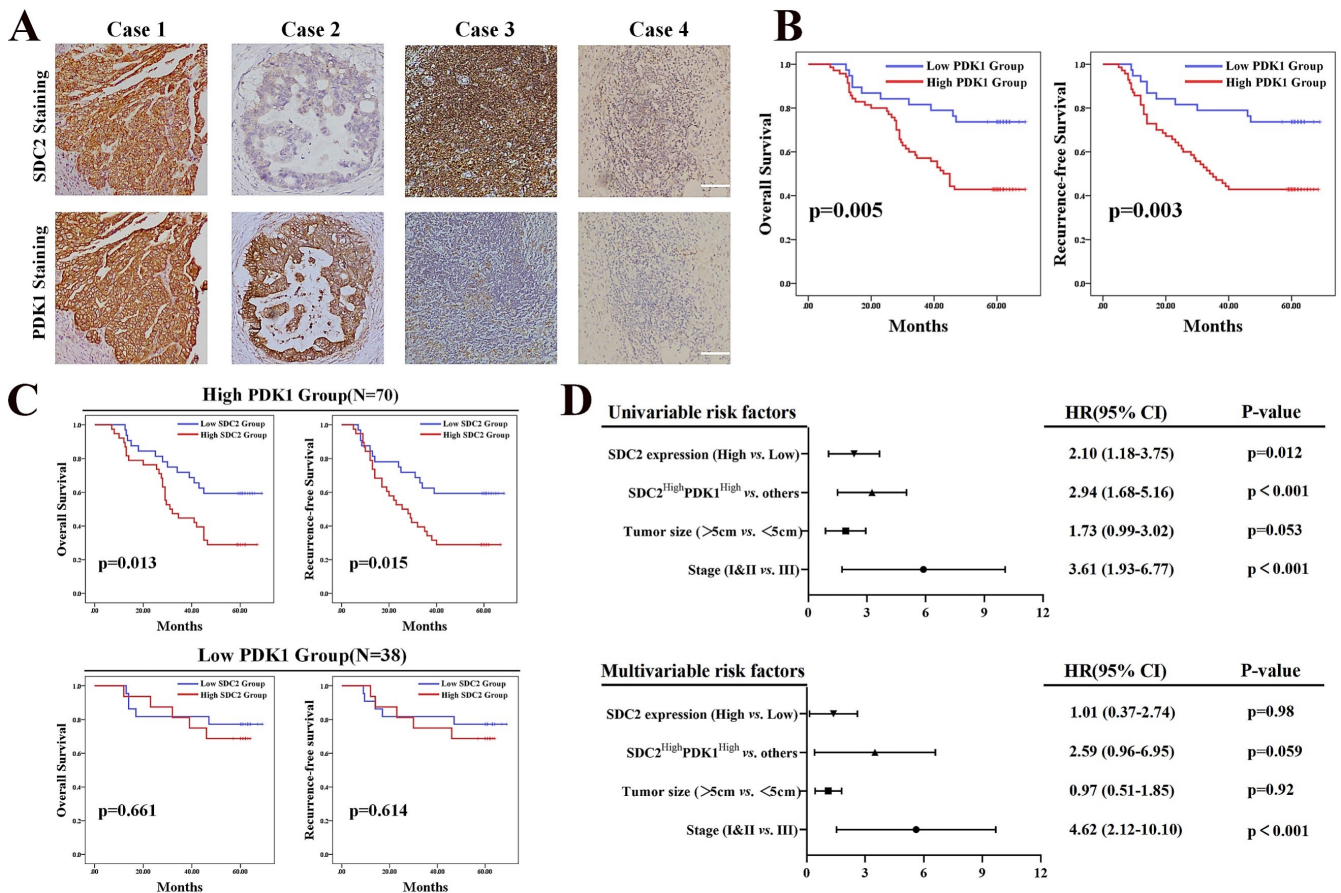
** High expression of SDC2 and PDK1 predicts poor postoperative survival of GC patients. A.** Representative GC tissues of four patients with different levels of SDC2 and PDK1 expression (1: SDC2^High^PDK1^High^, 2: SDC2^Low^PDK1^High^, 3: SDC2^High^PDK1^Low^, 4: SDC2^Low^PDK1^Low^) (Immunohistochemistry, scale bar: 100 µm).** B.** Overall survival, and recurrence-free survival of GC patients in the high PDK1 group (N = 70) and the low PDK1 group (N = 38) (Immunohistochemistry, FUSCC cohort). **C.** Overall survival and recurrence-free survival of GC patients who had high SDC2 expression or low SDC2 expression and were in the high PDK1 group (top, N = 70) or the low PDK1 group (bottom, N = 38) (FUSCC cohort). **D.** Univariate and multivariate Cox regression analysis of factors associated with post-operative survival time in patients with GC (FUSCC cohort).

Our results demonstrated that the upregulation of SDC2 correlated with GC severity and progression, and our investigation of the underlying mechanism showed that AKT pathway activation *via* a physical interaction with PDK1 played an important role in this process. It has been proven that PDK1 functions as an oncogene in GC 43. We thus investigated whether high expression of SDC2 and PDK1 indicates any synergistic oncological outcomes in GC patients. We hence performed IHC examination of SDC2 and PDK1 in our FUSCC in-house cohort, with classification of these patients into four different groups according to SDC2 and PDK1 expression: SDC2^High^PDK1^High^, SDC2^Low^PDK1^High^, SDC2^High^PDK1^Low^, and SDC2^Low^PDK1^Low^ GC tissues (**Fig. 5A**). GC patients with high expression of PDK1 (IHC score > 6) had significantly shorter OS and RFS than those with low expression (**Fig. 5B**). Thus, we divided these GC patients into PDK1^High^ and PDK1^Low^ subgroups. Kaplan-Meier analysis indicated that the SDC2^Low^PDK1^High^ group had a significantly longer OS and RFS than the SDC2^High^PDK1^High^ group; in contrast, the SDC2^High^PDK1^Low^ group and the SDC2^Low^PDK1^Low^ group had similar OS and RFS (**Fig. 5C**). These results support our findings as described above that the oncogenic effects of SDC2 in GC largely rely on PDK1. Furthermore, our univariable and multivariable Cox regression analysis of the FUSCC cohort showed that the status of SDC2^High^PDK1^High^ was an independent prognostic factor for poor postoperative survival time (**Fig. 5D**).

### SDC2 is involved in the FGF2-AKT signaling axis in GC cells

Previous studies demonstrated that SDC2 can function as a co-receptor for FGF2 (a heparin-binding growth factor), thus modulating its downstream signaling 31, 32, 44, 45. There is also evidence that SDC2 interacts with FGFR1, FGFR2, and/or FGFR3, the main receptors for FGF2 45-48. Together with the known role of FGF2 in the induction of the downstream PI3K-AKT signaling pathway 49-51 and inspired by our protein interaction analysis which revealed an interaction between FGF2 and SDC2 (**Fig. 4A and 4B**) and the SDC2-PDK1-mediated activation of the AKT pathway, we speculated whether SDC2 is also involved in the FGF2-AKT signaling axis in GC. To understand the biological effects induced by FGF2-SDC2 binding in GC cells, we added exogenous human FGF2 at a final concentration of 20 ng/mL or 100 ng/mL into the culture medium. In these experiments, cells were starved in low serum medium (2% FBS) overnight and then stimulated by FGF2 for 30 min as described in previous studies 9, 49. Our pilot experiment demonstrated that 100 ng/mL was an effective dose, and that SDC2 knockdown impaired the FGF2-induced phosphorylation of AKT in GC cells (**Fig. S4A**).

**Figure 6:**
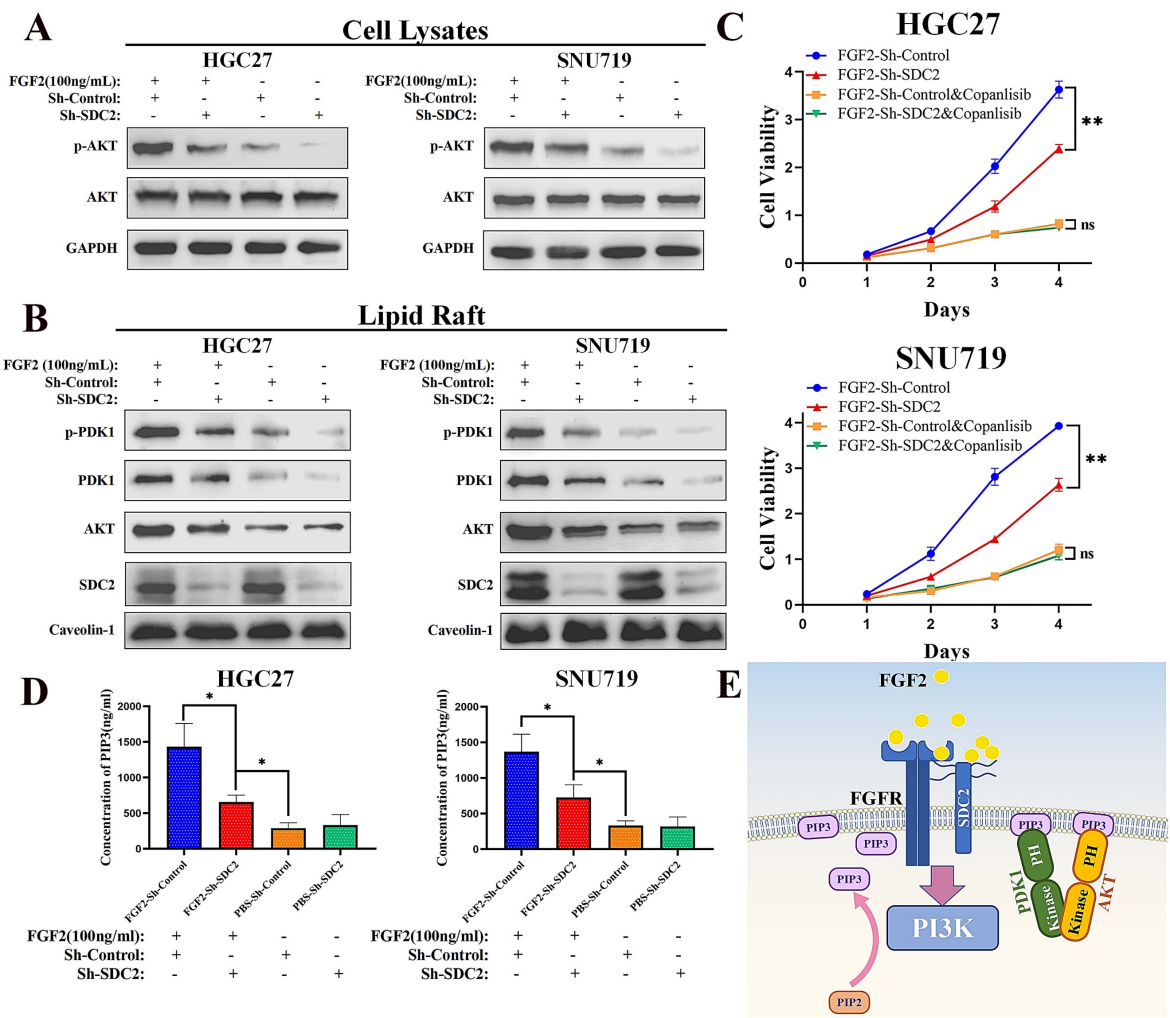
** SDC2 is involved in the FGF2-AKT signaling axis in GC cells. A&B.** Effect of FGF2 stimulation and SDC2 on the PI3K/AKT signaling pathway in two lines of GC cells (Western blotting).** C.** Effect of the FGF2-SDC2 binding on cell viability, and reversal of this effect by copanlisib in two lines of GC cells (CCK8 assay). **D.** Concentration of PIP3 in the plasma membrane in two lines of GC cells that received different treatments (ELISA).** E.** A schematic diagram showing the proposed mechanism.

When we performed similar experiments to examine the response to IGF1, the results showed that SDC2 had a much weaker influence in regulating the IGF1-AKT signaling axis in GC cells (**Fig. S4B**). Western blotting results showed that FGF2 stimulation upregulated the AKT pathway in GC cells (**Fig. 6A**) by increasing the membrane translocation of both PDK1 and AKT (**Fig. 6B**), and that knockdown of SDC2 impaired this effect. We next investigated how FGF2-SDC2 binding regulated the progression of GC. We therefore applied Copanlisib (a pan-PI3K inhibitor) to suppress PI3K-AKT signaling in cells. We first cultured HGC27 and SNU719 GC cells in different concentrations of copanlisib for 48 h to determine the most appropriate drug concentration for subsequent experiments (**Fig. S4C**). The results showed that FGF2-SDC2 binding increased the growth rate of GC cells, and this effect largely depended on the regulation of the PI3K-AKT signaling pathway (**Fig. 6C**). Collectively, these results indicated that FGF2-SDC2 binding promoted AKT phosphorylation by regulating canonical PI3K-AKT signaling. Because PI3K activation by cytokine receptors is very complicated, involving the regulation of two subunits and several variants 52, 53, we assessed the effect of PI3K kinase by measuring the PIP3 concentration in plasma membranes using ELISA, as described previously 54. The results showed that FGF2 stimulation clearly increased the PIP3 concentration in plasma membranes, and that SDC2 knockdown impaired this effect (**Fig. 6D**). Based on these results, we developed a mechanistic model that shows how SDC2 modulates the FGF2-AKT signaling axis in GC (**Fig. 6E**)**.**

### USP14 stabilizes SDC2 through reducing its ubiquitin-mediated degradation in GC cells

**Figure 7:**
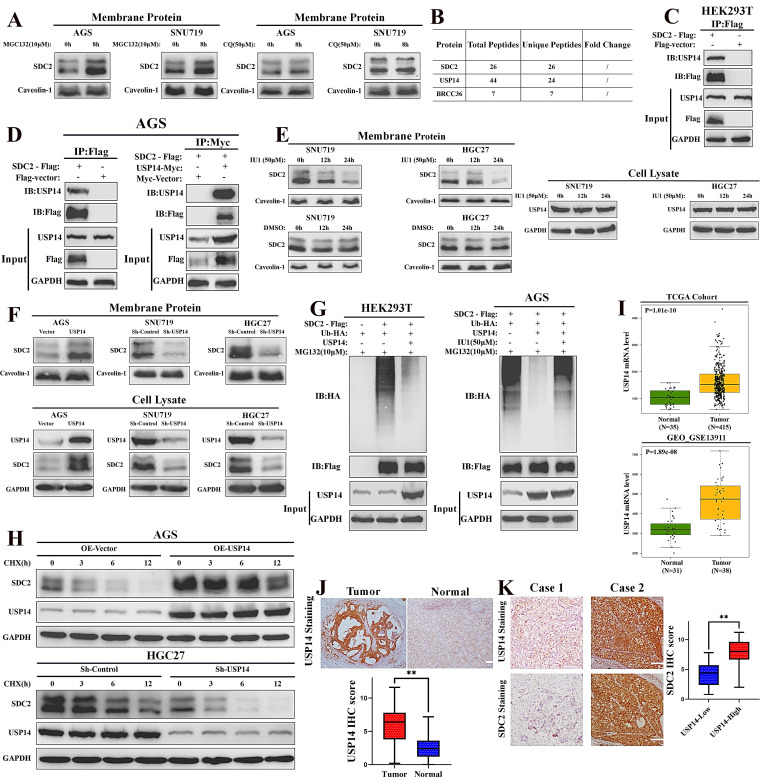
** USP14 stabilizes SDC2 by decreasing its ubiquitin-mediated degradation in GC cells. A.** Effect of two ubiquitin inhibitors (MG132 and chloroquine [CQ]) on the protein level of SDC2 in two lines of GC cells (Western blotting).** B.** USP14 and BRCC36 were probably associated with SDC2 (LC/MS). **C & D.** Interaction of SDC2 and USP14 in HEK 293T cells and AGS GC cells (Co-IP assay).** E.** Effect of IU1 on the level of SDC2 and USP14 in two lines of GC cells (Western blotting). **F.** Effect of transfection with different USP14 plasmids on the level of SDC2 in three lines of GC cells (Western blotting).** G.** Effect of transfection with different USP14 plasmids and treatment with IU1(24 h) on ubiquitination of SDC2. **H.** GC cells transfected with different USP14 plasmids were treated with CHX (0.1 mg/mL) for the indicated time. **I.** Levels of USP14 mRNA in GC tissues and adjacent normal tissues in two patient cohorts (TCGA, GEO-GSE).** J.** USP14 expression in GC tissues (N=78) and normal tissues (N=78) (Immunohistochemistry, Cases No. 31-108, FUSCC cohort, scale bar: 100 µm).** K.** SDC2 expression in GC tissues of the USP14^Low^ group (N=22) and the USP14^High^ group (N=56).

We next examined the possible mechanisms responsible for the high abundance of SDC2 in GC, and hypothesized whether this involves the ubiquitin-mediated regulation. Thus, we treated GC cells with different inhibitors of ubiquitin: MG132 (which inhibits the proteasomal degradation) and chloroquine (CQ, which inhibits the lysosomal degradation). The western blotting results showed that the ubiquitin (Ub)-mediated degradation of SDC2 was largely proteasome-dependent, in that CQ had little effect but MG132 led to an increased level of SDC2 (**Fig. 7A**). Our LC-MS analysis identified two de-ubiquitinases that were likely to affect SDC2: Ubiquitin Specific Peptidase 14 (USP14) and Lys-63-specific de-ubiquitinase (BRCC36) (**Fig. 7B and Fig. S5**). BRCC36 functions by stabilizing its substrate through antagonizing the K63 Ub-selective autophagy-lysosome pathway 55. Our results shows that inhibition of this pathway had little effect on SDC2 stability (**Fig. 7A**). We thus focused on the role of USP14 in maintaining the abundance of SDC2.

The Co-IP experiments verified an interaction of SDC2 with USP14 in HEK293T cells and AGS cells (**Fig. 7C and 7D**). Inhibition of USP14 by IU1 clearly decreased the protein level of SDC2 in SNU719 and HGC27 cells (**Fig. 7E**). We then manipulated the expression of USP14 using different plasmids and examined the abundance of SDC2 in both membrane protein and total protein. Western blotting revealed that knockdown of USP14 significantly decreased the protein level of SDC2, whereas overexpression of USP14 apparently increased the protein level of SDC2 in GC cells (**Fig. 7F**). The results of ubiquitination assays indicated that ectopic expression of USP14 significantly decreased the ubiquitination of SDC2, and that IU1 treatment reversed this effect (**Fig. 7G**). To thoroughly examine the role of USP14 in SDC2 protein, we performed a cycloheximide chase assay and found that overexpression of USP14 markedly prolonged the half-time of SDC2 protein, while silencing of USP14 exerted an opposite effect (**Fig. 7H**). These results demonstrate that USP14 stabilizes SDC2, and therefore might contribute to the progression of GC. Consistent with this hypothesis, we found that the USP14 mRNA levels were significantly higher in cancerous tissues than adjacent normal tissues in two independent cohorts of GC patients (**Fig. 7I**). IHC staining of USP14 in our FUSCC cohort showed that the IHC score was significantly higher in carcinoma tissues than normal tissues, and the expression of SDC2 was significantly higher in the High-USP14 group (IHC score > 4) than the Low-USP14 group of GC tumor specimens (**Fig. 7J and 7K**). Altogether, these results suggest a mechanism in which USP14.

## Discussion

The core protein of SDC2 consists of an extracellular domain to which several heparin sulfate (HS) chains are attached, a transmembrane (TM) domain, and a cytoplasmic domain (CD) which is composed of two conservative regions (C1 and C2) and one variable region 17. The HS chains of SDC2 consists of repeating disaccharide units of N-acetylglucosamine and D-glucuronic/iduronic acid 18, 56, and the sulfation modification of HS chains allow them to interact with specific growth factors 56. Furthermore, different SDC proteins can have a unique TM domain 17. The TM and the CD of SDC2 can interact with a wide range of proteins, resulting in a diversity of biological functions in various cells 17, 56. Previous studies have described several signaling pathways related to the oncogenic effects of SDC2 in multiple types of tumors. For example, SDC2 contributed to the malignancy of pancreatic cancer cells by regulating the K-ras/MAPK pathway 24, and enhanced the invasiveness of lung adenocarcinoma by upregulating the NF- κB signaling pathway 27. In squamous cell carcinoma, high expression of SDC2 was correlated with the activation of the AKT pathway, but the underlying molecular mechanisms remained unknown 57. Our study is the first to show that SDC2 functions as a potent oncogene in GC, in that it increases GC malignancy by enhancing PI3K/AKT signaling.

The results of our Co-IP assays and LC-MS analysis led to identification of a protein-protein interaction between SDC2 and PDK1. PIP3 is a well-established membrane anchor for PDK1 11. Moreover, it has been demonstrated that some other membrane proteins, such as AMIGO2, can also facilitate the membrane localization of PDK1, thereby modulating downstream signaling pathways 58-61. Park et al. demonstrated that AMIGO2 can enhance PI3K/AKT pathway through recruiting PDK1 to the plasma membrane via the formation of an AMIGO2-PIP3-PDK1 complex 58. The results of our Co-IP and molecular docking analyses indicated that SDC2 could directly bind with the PH domain of PDK1, thereby promoting the membrane translocation of PDK1. But the detailed structural mechanisms underlying the protein-protein interaction between SDC2 and PDK1-PH domain require more research.

Another important finding in our study is that SDC2 also interacts directly with FGF2, and this also regulates the PI3K-AKT axis in GC cells. Several possible mechanisms may be responsible for this effect. First, SDC2 could protect and concentrate FGF2 on the membrane surface using its HS chains 18, 45. Second, the HS chains of SDC2 might promote the dimerization of FGF2, thus facilitating its presentation to its receptors 47. Third, SDC2 might interact with the high-affinity receptors of FGF2, including FGFR1, FGFR2, and/or FGFR3, and thereby increase their affinity for FGF2 17, 46, 48, 62. Our experimental results indicated that the binding of FGF2 with SDC2 enhanced the activation of PI3K, resulting in an increased plasma membrane level of PIP3 (the direct product of PI3K), which then functioned as the membrane anchor for PDK1 and AKT.

Ub-mediated regulation of key oncogenes plays an important role in tumor progression, and many recent studies examined ubiquitination as a potential target in cancer therapies 33, 34, 63, 64. Carvallo et al. found that SDC4 (in the same family as SDC2) was degraded in a proteasome-dependent manner 65. SDC2 has 3 predicted ubiquitination sites (lysine 185, 193, and 197) according to The Protein Lysine Modification Database. In future studies, we plan to identify the specific role of each site in the ubiquitin-mediated degradation of SDC2. USP14 is an important de-ubiquitinase, in that it protects various cytoplasmic and membrane proteins from ubiquitin-mediated degradation 35, 64, 66, 67. USP14 has two domains, a ubiquitin-like domain at its N-terminus and a catalytic domain that contains three active sites (C114, H435, and D451) at its C-terminus 35, 67. USP14 antagonizes E3 ligases and stabilizes its substrates, thereby preventing proteasomal degradation through removal of the attached ubiquitin chains 67. Our LC-MS analysis indicated that several E3 ligases, including RNF168, RING2, and TRIM25, may be associated with SDC2. USP14 antagonized RNF168-dependent proteasomal degradation 68, but its effect on other E3 ligases requires further investigation. Considering the pathogenic effects of USP14 in cancer and other diseases, several studies have described the development of small molecule inhibitors (SMI) that target USP14 69-71. For example, IU1 binds to the catalytic region of USP14 and is one of the most effective SMIs for suppression of USP14 activity 71. Lv et al. showed that IU1 decreased the protein level of HIF1-α, a USP14-mediated oncogene that functions in hepatocellular carcinoma 70. Likewise, our results demonstrated that addition of IU1 to GC cells for 24 h significantly reduced the abundance of SDC2. Thus, modulation of protein degradation by targeting the ubiquitin-proteasome pathway is a strategy that has potential for the indirect targeting of oncogenes that are difficult to directly target using conventional methods.

In conclusion, our study revealed that SDC2 functioned as a potent oncogene in GC, in that it promoted GC cell growth and invasiveness by upregulating the PI3K-AKT signaling pathway. Our investigation of the mechanism indicated that SDC2 interacted with the PH domain of PDK1, thereby facilitating its membrane translocation and upregulation of the AKT pathway. Moreover, as an effective co-receptor for FGF2, SDC2 enhanced the FGF2-AKT signaling axis in GC cells. Because USP14 deubiquitinated and stabilized SDC2 in GC, this suggests the feasibility of a therapeutic strategy that decreases the abundance of SDC2 by using USP14-specific inhibitors, such as IU1.

However, this study still had several limitations. Firstly, in addition to the USP14-mediated stabilization of SDC2 at the protein level, we cannot rule out the possibility that the aberrant transcriptional activation mechanism led to increased SDC2 expression and thus protein translation, which warrants further investigations. Secondly, the details of the protein structure of SDC2's cytoplasmic domain have not yet been resolved, so we were unable to evaluate the precise nature of the protein-protein docking between the entire SDC2 protein and the PH domain of PDK1. Thirdly, we did not directly demonstrate the activation of PI3K kinase using western blotting; instead, we used ELISA to measure PIP3 membrane concentration to assess the state of PI3K. Membrane proteins, particularly those that oligomerize in lipid rafts, have shown some resistance to detergents 72. Therefore, we opted to use membrane proteins or lipid rafts instead of cell lysates for western blotting measurements of SDC2.

## Supplementary Material

Supplementary figures and tables.

## Figures and Tables

**Figure 8:**
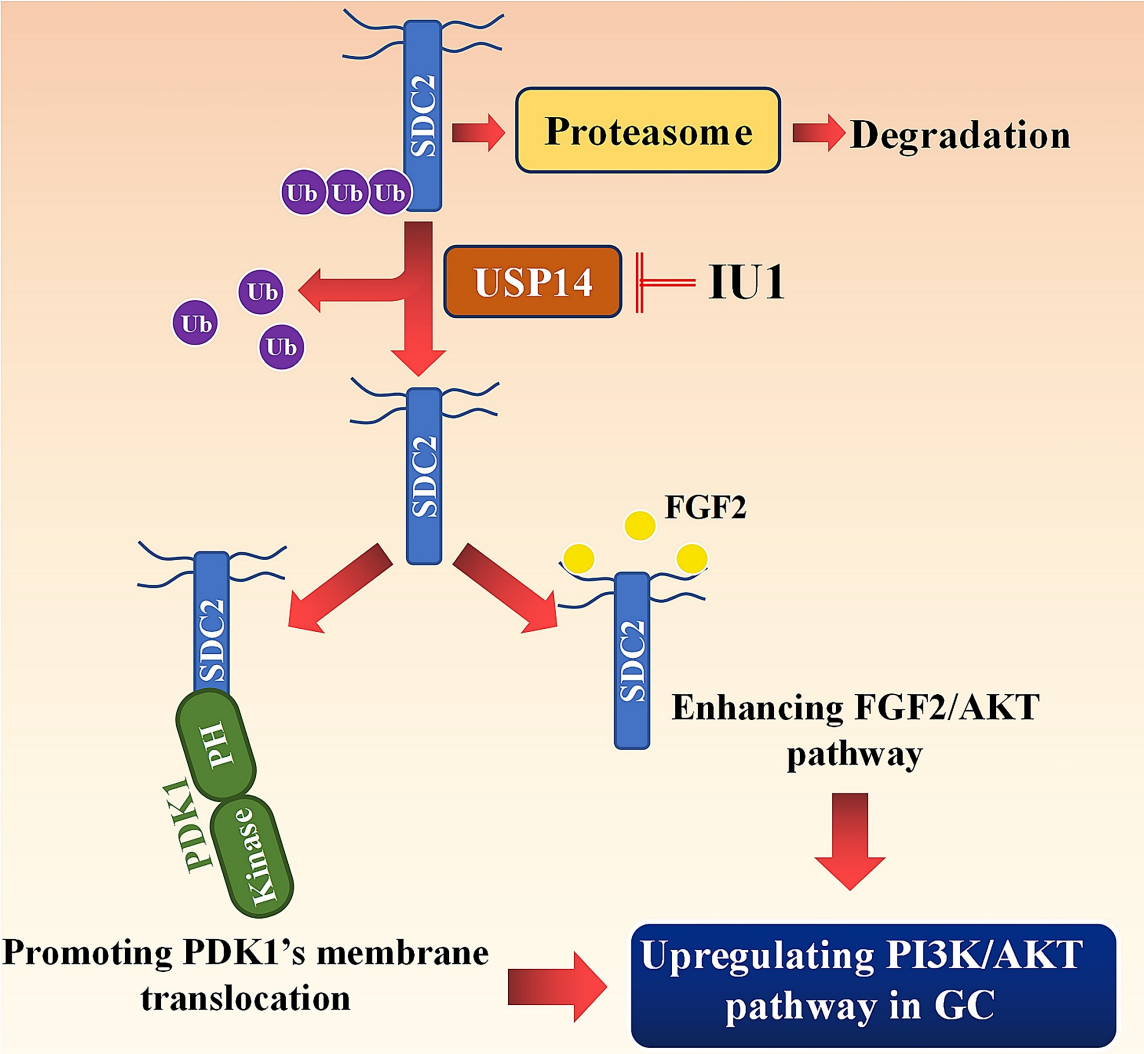
** Working model:** SDC2 promotes gastric cancer progression through co-option of PDK1 and modulating the FGF2-AKT signaling axis***.*** USP14 deubiquitinates and stabilizes SDC2, while IU1 treatment decreases the abundance of SDC2 through targeting USP14.

## Abbreviations

GC: Gastric cancer
SDC2: Syndecan 2
PDK1: 3-Phosphoinositide dependent protein kinase 1
AKT: Protein kinase B
PH domain: Pleckstrin-homology domain
TM domain: Transmembrane domain
FGF2: Fibroblast growth factor 2
PIP3: Phosphatidylinositol trisphosphate
Ub: Ubiquitin
USP14: Ubiquitin specific peptidase 14
GO: Gene Ontology
TCGA: The Cancer Genome Atlas
GEO: Gene Expression Omnibus
KEGG: Kyoto Encyclopedia of Genes and Genomes
Elisa: Enzyme-linked Immunosorbent assay
EMT: Epithelial-mesenchymal transition
OS: Overall survival
RFS: Recurrence-free survival
