# Supplementary material for: SDC2 Stabilization by USP14 Promotes Gastric Cancer Progression through Co-option of PDK1
Source: Int J Biol Sci. 2023 Jul 9;19(11):3483–98. doi: 10.7150/ijbs.84331 (PMC10367555; doi:10.7150/ijbs.84331)
Supplement: Supplementary file 1 — Supplementary figures and tables. [file ijbsv19p3483s1.pdf]

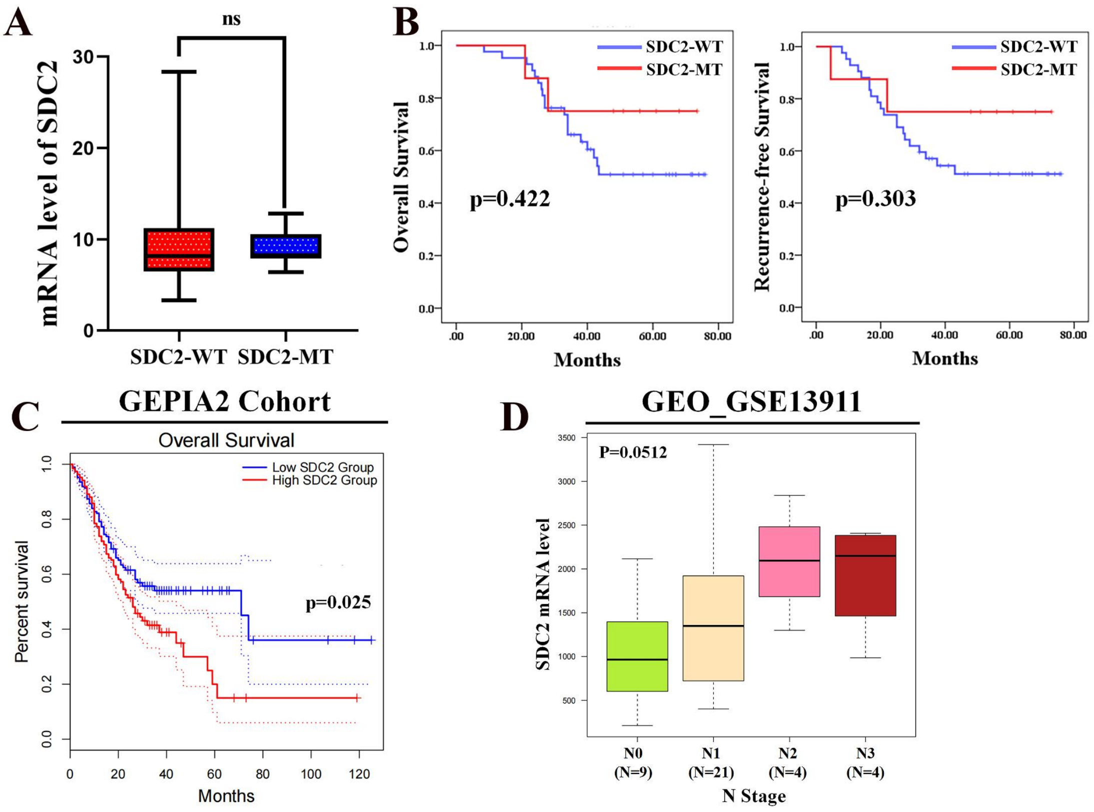

Figure S1.

A. SDC2 mRNA levels in the GC tissues of patients in the SDC2-WT group (N = 42) and the SDC2-MT group (N = 8) (SYSUCC Cohort). **B.** Overall survival and recurrence-free survival of GC patients in the SDC2-WT group (N = 42) and the SDC2-MT group (N = 8) (SYSUCC Cohort). **C.** Overall survival of GC patients with high SDC2 expression (N = 192) and low SDC2 expression (N = 192) (GEIPA2 Cohort). **D.** SDC2 mRNA levels in GC tissues with different N stages (GES13911 Cohort).

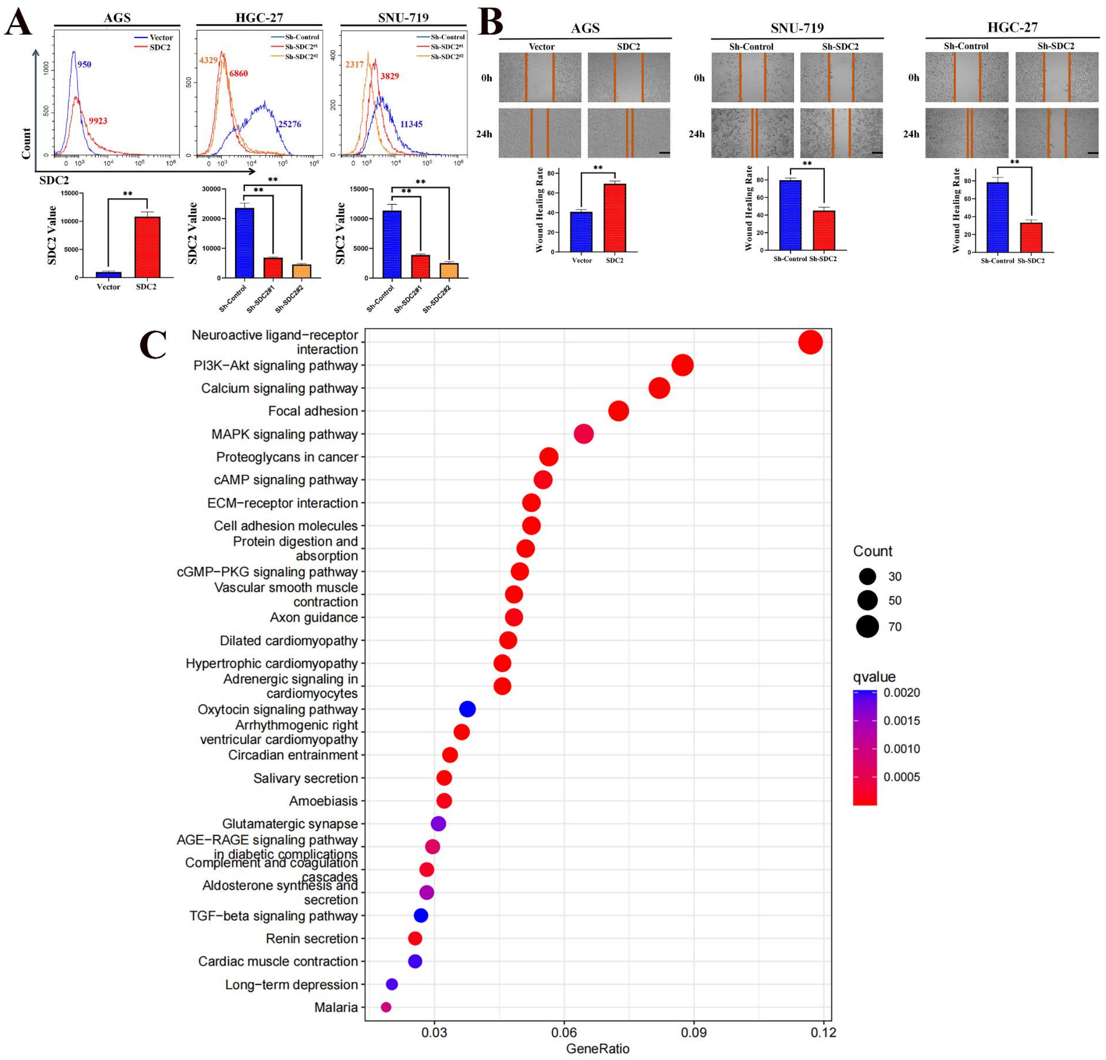

Figure S2.

A. Protein levels of SDC2 in three lines of GC cells after stable transfection with different SDC2 plasmids was measured using flow cytometry. **B.** Cell migration of AGS, SNU719, HGC27 cells that were transfected with different plasmids (Wound healing assay, scale bar: 200 µm). **C.** Association of SDC2 with enrichment of signaling pathways (KEGG enrichment using the TCGA database).

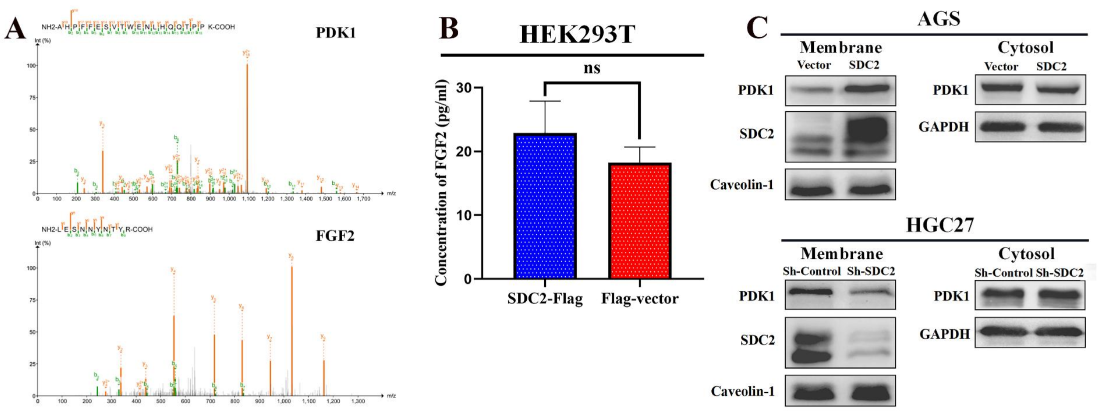

Figure S3.

A. Peptide mapping of PKD1 and FGF2 (LC/MS). **B.** Concentration of FGF2 in culture medium of 293T cells transfected with SDC2-Flag or Flag-vector plasmids. **C.** Effect of SDC2 on membrane translocation of PDK1 in AGS and HGC27 cells (Western blotting).

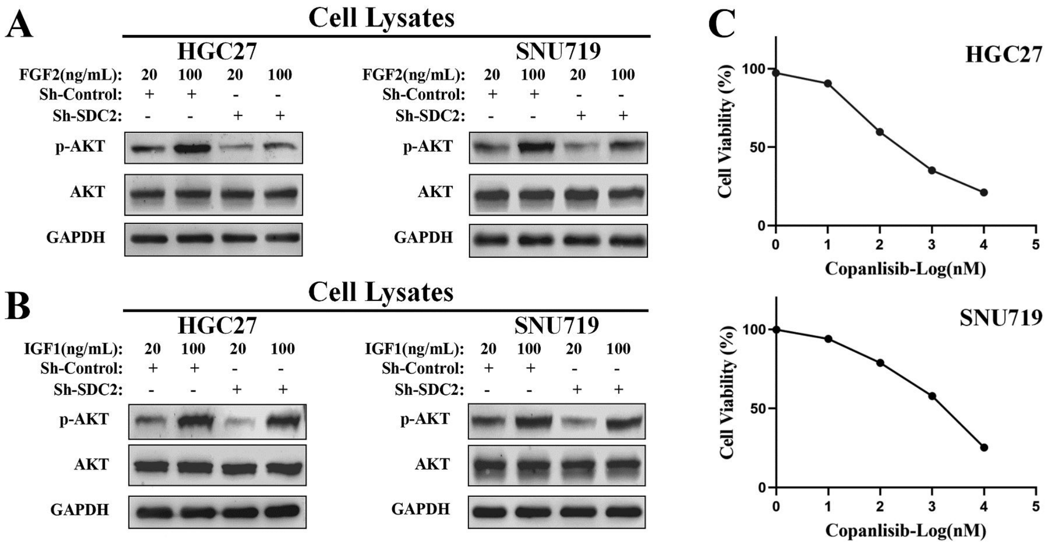

Figure S4.

A & B. FGF2-induced and IGF1-induced phosphorylation of AKT in GC cells with or without SDC2 knockdown (Western blotting). **C.** Effect of different Copanlisib concentration on the viability of two GC cell lines (treatment for 48 h).

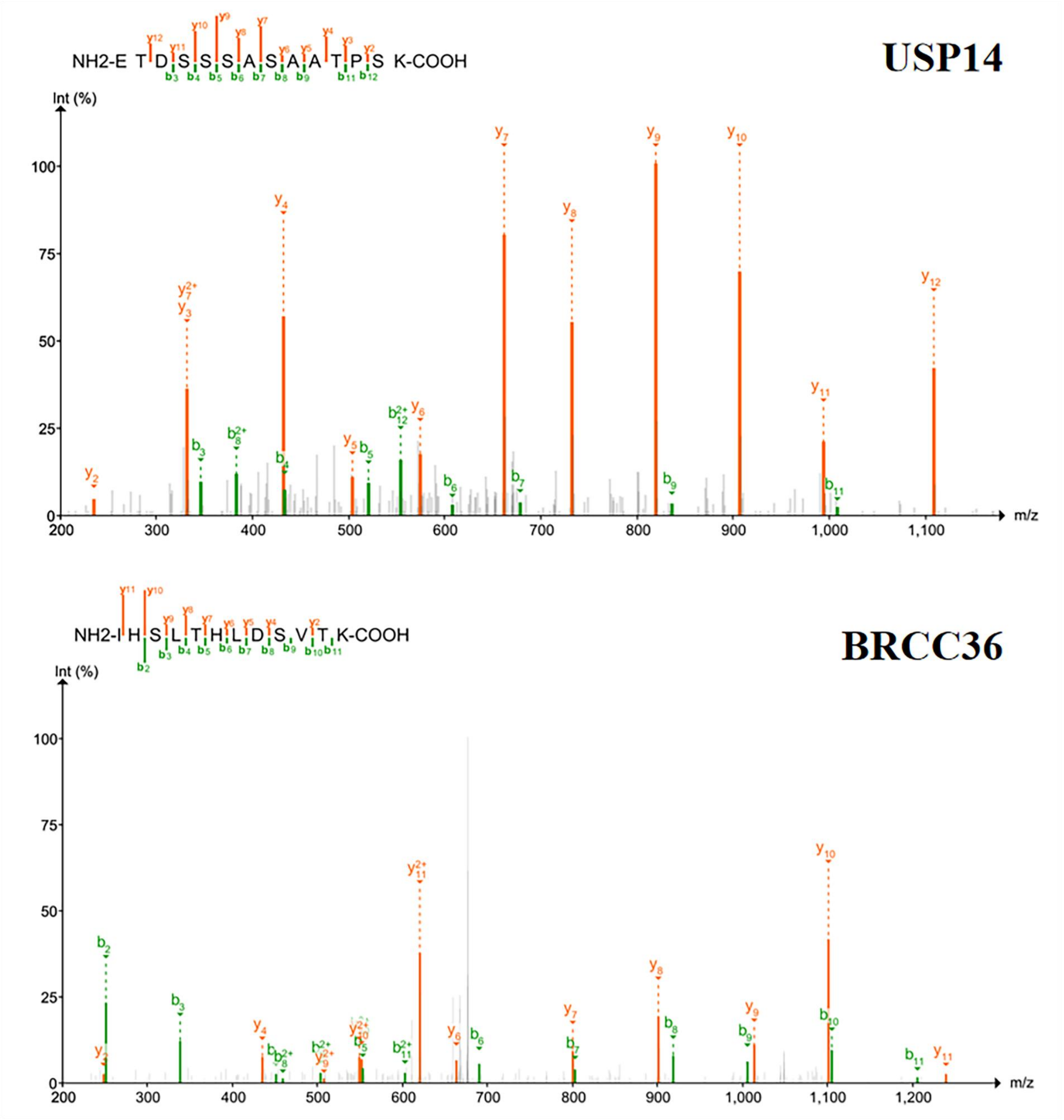

Figure S5.

Peptide mapping of USP14 and BRCC36 (LC/MS).

Table S1. Information for plamids used in this study

shRNAs	Sequences
Sh-SDC2[#1]	5'- GACATGTACCTTGACAACAGC -3'
Sh-SDC2[#2]	5'- GCTTCAGGAGTGTATCCTATT -3'
Sh-PDK1	5'- CAACATAGAGCAGTACATTCA -3'
Sh-USP14	5'- CCCAAGATTCAGCAGTCAGAT -3'

Overexpression plasmids	NM number
Lenti-CMV-H_SDC2-3×Flag-PGK-Puro	NM_002998
PGMLV-CMV-H_PDPK1-HA-PGK-Blasticidin	NM_002613
PGMLV-CMV-H_USP14-PGK-Puro	NM_005151
Lenti-CMV-H_USP14-myc-PGK-Blasticidin	NM_005151

Table S2. Information for WB antibodies used in this study

	**Identifiers**	**Source**
SDC2	*# 365624*	Santa Cruz, USA
PDK1	*# 13037*	Cell Signaling Technology, USA
p-PDK1	*# 3438*	Cell Signaling Technology, USA
AKT	*# 4691*	Cell Signaling Technology, USA
p-AKT	*# 4060*	Cell Signaling Technology, USA
GAPDH	*# 5174*	Cell Signaling Technology, USA
Caveolin-1	*# 3267*	Cell Signaling Technology, USA
N-cadherin	*# 13116*	Cell Signaling Technology, USA
Vimentin	*# 46173*	Cell Signaling Technology, USA
FGF2	*# 98658*	Cell Signaling Technology, USA
USP14	*# 11931*	Cell Signaling Technology, USA
HA	*# 3724*	Cell Signaling Technology, USA
Flag	*# 66008-4-Ig*	Proteintech, China

Table S3. Correlation between SDC2-mutation and clinicopathological characteristics of GC patient samples (using Cohort 1-SYSUCC Cohort)

Characteristics	SDC2-WT, No. of cases	SDC2-MT, No. of cases	*P* value
**Age(y)**			
<60	24	5	0.778
≥60	18	3	
**Gender**			
Male	39	7	0.609
Female	3	1	
**Tumor Location**			
Proximal	10	4	0.051
Middle	25	1	
Distal	7	3	
**Tumor Size**			
<5 cm	22	5	0.599
≥5 cm	20	3	
**TNM stage**			
I	5	0	0.400
II	10	4	
III	26	4	
IV	1	0	

Table S4. Correlation between SDC2 mRNA level and clinicopathological characteristics of GC patient samples (using Cohort 1-SYSUCC Cohort)

Characteristics	SDC2 mRNA level		*P* value
	Low expression, No. of cases	High expression, No. of cases	
**Age(y)**			
<60	12	17	0.152
≥60	13	8	
**Gender**			
Male	24	22	0.297
Female	1	3	
**Tumor Location**			
Proximal	9	5	0.415
Middle	11	15	
Distal	5	5	
**Tumor Size**			
<5 cm	15	12	0.395
≥5 cm	10	13	
**TNM stage**			
I	3	2	0.686
II	6	8	
III	15	15	
IV	1	0	

Table S5. Correlation between SDC2 IHC expression level and clinicopathological characteristics of GC patient samples (using Cohort 2-FUSCC Cohort)

Characteristics	SDC2 IHC expression level		*P* value
	Low expression, No. of cases	High expression, No. of cases	
**Age(y)**			
<60	22	27	0.334
≥60	32	27	
**Gender**			
Male	39	34	0.304
Female	15	20	
**Tumor Location**			
Proximal	23	28	0.335
Distal	31	26	
**Tumor Size**			
<5 cm	40	31	0.068
≥5 cm	14	23	
**TNM stage**			
I	10	6	0.556
II	13	14	
III	31	34	
